# Ice Water

**Published:** 1860-08

**Authors:** 


### ICE-WATER.

If the reader is down-town or away from home on a hot day, and feels as if it would be perfectly delicious to have a glass of lemonade, soda-water, or brandy toddy, by all means let him resist the temptation until he gets home, and then take a glass of cool water, a swallow at a time, with a second or two interval between each swallow. Several noteworthy results will most assuredly follow.

After it is all over, you will feel quite as well from a drink of water as if you had enjoyed a free swig of either of the others.

In ten minutes after you will feel a great deal better.

You will not have been poisoned by the lead or copper which is most generally found in soda-water.

You will be richer by six cents, which will be the interest on a dollar for a whole year!

You will not have fallen down dead from the sudden chills which sometimes result from drinking soda, iced water, or toddy, in a hurry.

No well man has any business to eat ices or to drink iced liquids in any shape or form, if he wants to preserve his teeth, protect the tone of his stomach, and guard against sudden inflammations and prolonged dyspepsias. It is enough to make one shudder to see a beautiful young girl sipping scalding coffee or tea at the beginning of a meal, and then close it with a glass of ice-water; for at thirty she must either be snaggle-toothed or wear those of the dead or artificial.

Fresh spring or well-water is abundantly cool for any drinking purpose whatever. In cities where water is artificially supplied, the case is somewhat different; but even then there is no good excuse for drinking ice-water, because, even if the excuse were good in itself, the effects on the stomach and teeth are the same.

Make a bag of thick woolen doubled, lined with muslin; fill it with ice; have in a pitcher an inch or two of water above the faucet, and let this bag of ice be suspended from the cover within two inches of the surface of the water. The ice will melt slowly and keep the water delightfully cool, but not ice cold. A still better effect will be produced if the pitcher is also well enveloped in woolen. Again, water almost as cool as it can be, unless it has ice actually in it, may be had without any ice at all, by enveloping a closed pitcher partly filled with water, with several folds of cotton, linen, or bagging, and so arranging it that these folds are kept wet all the time by water dripping from another vessel, on the principle of evaporation.

As to the management of refrigerators, Bartlett's "Polar," being the most philosophical, the ice never comes in contact with the provisions, (for freezing disorganizes all meats and vegetables,) hence can not freeze them, but keeps them just above the freezing point, dry and cool, while the arrangement is such, that the ice as it melts, is used for drinking purposes. Thus, six cents' worth of ice a day, or about twenty pounds, is, with proper management, enough for the use of a family of ten persons throughout the summer, not only for drinking purposes, but for the preservation of the eatables.

